# Novel Strategies for Enhanced Removal of Persistent *Bacillus anthracis* Surrogates and *Clostridium difficile* Spores from Skin

**DOI:** 10.1371/journal.pone.0068706

**Published:** 2013-07-02

**Authors:** Michelle M. Nerandzic, Elze Rackaityte, Lucy A. Jury, Kevin Eckart, Curtis J. Donskey

**Affiliations:** 1 Research Service, Cleveland Veterans Affairs Medical Center, Cleveland, Ohio, United States of America; 2 Geriatric Research, Education and Clinical Center, Cleveland Veterans Affairs Medical Center, Cleveland, Ohio, United States of America; 3 School of Medicine, Case Western Reserve University, Cleveland, Ohio, United States of America; University of Connecticut, United States of America

## Abstract

**Background:**

Removing spores of *Clostridium difficile* and *Bacillus anthracis* from skin is challenging because they are resistant to commonly used antimicrobials and soap and water washing provides only modest efficacy. We hypothesized that hygiene interventions incorporating a sporicidal electrochemically generated hypochlorous acid solution (Vashe^®^) would reduce the burden of spores on skin.

**Methods:**

Hands of volunteers were inoculated with non-toxigenic *C. difficile* spores or *B. anthracis* spore surrogates to assess the effectiveness of Vashe solution for reducing spores on skin. Reduction in spores was compared for Vashe hygiene interventions versus soap and water (control). To determine the effectiveness of Vashe solution for removal of *C. difficile* spores from the skin of patients with *C. difficile* infection (CDI), reductions in levels of spores on skin were compared for soap and water versus Vashe bed baths.

**Results:**

Spore removal from hands was enhanced with Vashe soak (>2.5 log_10_ reduction) versus soap and water wash or soak (~2.0 log_10_ reduction; *P* <0.05) and Vashe wipes versus alcohol wipes (*P* <0.01). A combined approach of soap and water wash followed by soaking in Vashe removed >3.5 log_10_ spores from hands (*P* <0.01 compared to washing or soaking alone). Bed baths using soap and water (N =26 patients) did not reduce the percentage of positive skin cultures for CDI patients (64% before versus 57% after bathing; *P* =0.5), whereas bathing with Vashe solution (N =21 patients) significantly reduced skin contamination (54% before versus 8% after bathing; *P* =0.0001). Vashe was well-tolerated with no evidence of adverse effects on skin.

**Conclusions:**

Vashe was safe and effective for reducing the burden of *B. anthracis* surrogates and *C. difficile* spores on hands. Bed baths with Vashe were effective for reducing *C. difficile* on skin. These findings suggest a novel strategy to reduce the burden of spores on skin.

## Introduction

Spores produced by the bacterial species *Clostridium difficile* and *Bacillus anthracis* are a formidable challenge to control because they are highly resistant to many chemical and physical agents and can persist for prolonged periods on skin and environmental surfaces [1]. *C. difficile* is the most common cause of healthcare-associated diarrhea in developed countries and has increased in ubiquity and virulence in the past decade [2,3]. Patients with *C. difficile* infection (CDI) shed spores in stool, resulting in contamination of their skin, clothing, and nearby environmental surfaces [4]. Healthcare workers’ hands serve as a vector for transmission of spores from these sites to susceptible patients [5]. Because spores persist long after the cessation of disease, CDI outbreaks are challenging to control and have often required sequential implementation of multiple control measures [6,7].

*B. anthracis* is acknowledged as an agent for bioterrorism because of its highly resistant and stable spores [8]. Exposure to *B. anthracis* spores causes anthrax, an infection associated with substantial morbidity and mortality [8]. Humans can contract anthrax through skin contact, ingestion, or inhalation of *B. anthracis* spores [8,9]. Intentional exposure to *B. anthracis* spores caused 22 people in the United States to develop anthrax after a bioterrorism event in 2001 [10,11].

Current guidelines to prevent dissemination of spore mediated diseases recommend decontamination of exposed individuals and environmental surfaces [12–15]. While there are disinfection methods to effectively address environmental contamination, there are currently no highly effective methods to reduce levels of spores on skin [12,13,16,17]. Procedures designed for effective sterilization of spores on environmental surfaces are harsh and cannot be safely applied to skin. Standard hand hygiene and bathing products, such as alcohol-based formulations and chlorhexidine gluconate, are not sporicidal [5,18,19]. Soap and water handwashing is currently the gold-standard for removing spores from hands and is indicated when contact with spores is suspected or likely [20]. However, soap and water handwashing is only moderately effective and has been reported to remove approximately 2 log_10_ colony forming units (CFU) of spores or less from hands [5,18,21,22]. Similarly, bed baths using soap and water did not significantly reduce levels of spores on the skin of patients with CDI [23]. Because effective hand hygiene and bathing interventions have been shown to reduce infections with other pathogens, there is an important unmet need for development of more effective strategies to reduce the burden of spores on skin [24–26].

Electrochemically activated saline solutions are broad spectrum disinfectants that are generated by passing saline solution through an electrolytic cell, resulting in production of hypochlorous acid and free radicals [27–29]. These solutions are commonly used for wound therapy, decontamination of dental water lines, high level disinfection of heat-sensitive medical devices, and washing fruits and vegetables [27–29]. Moreover, electrochemically activated saline solutions containing 0.025% to 0.05% hypochlorous acid (Vashe^®^ and Sterilox^®^, PuriCore, Malvern, PA) have been shown to have sporicidal activity against several species of bacillus and clostridia, including *C. difficile* [27]. In contrast to other sporicidal disinfectants, electrochemically activated saline solutions are safe for application on skin, and Vashe solution is commercially available as a wound care product. Here, we tested the hypothesis that hygiene interventions that include electrochemically activated saline solutions would effectively reduce the burden of spores on hands and on patients’ skin.

## Materials and Methods

### Ethics Statement

The Cleveland VA Medical Center’s Institutional Review Board approved the study protocol and all participants provided oral informed consent. The Institutional Review

Board approved the use of an oral consent because the study was considered minimal risk. An oral consent script was read to potential participants and they were given an opportunity to ask questions. After explaining the research, participants were asked a formal consent question: Do you provide your consent to participate in this study? For those agreeing to participate, the researcher recorded the subjects name in a study log book and documented that they had responded “Yes” when asked if they provided consent to participate in the study. In addition, the patients provided a signed Health

Insurance Portability and Accountability Act form which documented their participation.

### Setting and Participants

The Cleveland Veterans Affairs Medical Center is a 214-bed acute care hospital with an adjacent long-term care facility. All inpatients diagnosed with CDI during a 5 month study period were considered for the evaluation of bathing to remove spores from skin. CDI was diagnosed on the basis of the presence of diarrhea and a polymerase chain reaction test positive for *C. difficile* toxin B genes (*tcdB*) by a commercial assay (Xpert *C. difficile*, Cepheid, Sunnyvale, CA). In addition, 6 healthy research laboratory workers with no dermatologic diseases affecting their hands participated in hand wash studies.

### *Clostridium difficile* Strains and Growth Conditions

A restriction endonuclease analysis (REA) type BI epidemic isolate from the Cleveland VA Medical Center was used for experiments assessing the impact of organic load on disinfection of spores on surfaces. Two non-toxigenic strains of *C. difficile* from American Type Culture Collection (ATCC 43593 and 43601) were used for hand washing experiments. Cultures were incubated at 37 °C for 48 hours in a Whitley MG1000 anaerobic workstation (Microbiology International, Frederick, MD) on pre-reduced cycloserine-cefoxitin- *Brucella* agar containing 0.1% taurocholic acid and lysozyme 5 mg/mL (CDBA) [30]. For isolates obtained from sampling patient’s skin, *C. difficile* was confirmed on the basis of typical odor and appearance of colonies and by a positive reaction using *C. difficile* latex agglutination (Microgen Bioproducts, Camberly, UK).

### Preparation of *C. difficile* Spores

Spores were prepared by growth on brain-heart infusion agar (Becton Dickinson, Cockeysville, MD) supplemented with yeast extract (5 mg/ml) and L-cysteine (0.1%) at 37°C under anaerobic conditions as previously described [31]. Spores were stored at 4 °C in sterile distilled water until use. Prior to testing, spore preps were confirmed by phase contrast microscopy and malachite green staining to be > 99% dormant, bright-phase spores.

### *Bacillus anthracis* Surrogates and Growth Conditions

Three phylogenetic relatives of *B. anthracis* previously identified as experimental surrogates were used for hand hygiene experiments [21,32,33]. *Bacillus* *atrophaeus* spore suspensions were purchased from NAMSA (Northwood, Ohio, USA). *Bacillus thuringiensis* and *Bacillus subtilis* spore suspensions were purchased from Mesa Labs (Omaha, Nebraska, USA). *B* *. atrophaeus* was chosen as a suitable surrogate for experiments assessing the impact of organic load because it has been reported as less susceptible to disinfecting agents than *B. anthracis* [21]. Spore suspensions were stored at 4 °C and diluted in sterile deionized water before use. Prior to testing, spore suspensions were confirmed by phase contrast microscopy and malachite green staining to be > 99% dormant, bright-phase spores. Cultures were incubated aerobically at 37 °C for 48 hours on trypticase soy agar containing 5% sheep blood.

### Impact of Organic Load on the Efficacy of Electrochemically Activated Saline

Because organic material may reduce the efficacy of disinfectants on surfaces or skin, initial experiments were conducted to examine the impact of organic load on efficacy of Sterilox solution for killing *C. difficile* and *B* *. atrophaeus* spores inoculated onto surfaces from patient rooms (i.e. bedrails, call buttons, and bedside tables). Sterilox was chosen for these experiments rather than Vashe, because Sterilox is commonly used for instrument or surface disinfection. For these experiments, the surfaces tested were transferred to the laboratory. Spores were suspended in decreasing dilutions of a simulated organic load consisting of bovine serum albumin (BSA), tryptone, and mucin suspended in deionized water. Ten µl aliquots of the spore suspensions (6 log_10_ colony-forming units (CFU)) were inoculated onto surfaces, spread to cover a 1 cm^2^ area, and allowed to air dry. Once dry, the contaminated surfaces were sprayed with deionized water (control) or Sterilox solution; 10% household bleach solution (Clorox, Oakland, California) was included at the highest level of organic load and in the absence of organic load to provide a comparison with a standard sporicidal surface disinfectant. After 5 minutes of contact time with the solutions, cultures were collected using sterile swabs (BD BBL™ CultureSwab™, Becton Dickinson, Cockeysville, MD) that were immersed in 1 ml of Dey-Engley neutralizer (Remel Products, Lenexa, KS). The swabs were cultured as described previously. Following 48 hours incubation, colonies of *C. difficile* and *B* *. atrophaeus* were counted using Fotodyne’s TotalLab Quant Analysis software (Fotodyne Inc., Hartland, Wisconsin). Log_10_ reduction was calculated by subtracting log_10_ CFU recovered after Sterilox or bleach treatment from log_10_ CFU recovered after water treatment (baseline control). The experiments were repeated four times.

### Randomization and Masking

For the hand disinfection experiments, a crossover design was used such that each of the 6 volunteers was exposed to all of the disinfection procedures. The order of the hand disinfection procedures for each volunteer was assigned using a computer-generated random numbers list. The person reading the plates to quantify spore counts was blinded to the test product that was used. For the bed bath experiments involving patients, the treatment groups were not randomly assigned because fresh Vashe solution was not consistently available. For these experiments, all consenting patients were assigned to the Vashe group during periods when fresh product was available.

### Effectiveness of Vashe for Removal of Spores from Hands

The Standard Test Method for Evaluation of the Effectiveness of Health Care Professional Handwash Formulations (American Society for Testing and Materials E 1174-06) was used [34]. Preliminary experiments demonstrated that hand disinfection with Vashe and Sterilox solutions yielded similar reductions in spores; Vashe was chosen as the primary product for subsequent studies because it is approved by the Food and Drug Administration (FDA) for use in wounds. Preliminary experiments also suggested that, due to the aqueous nature of Vashe and Sterilox, application was most effective when applied with a terry cloth saturated with the solutions or when hands were submersed and soaked in the solutions.

Hands were contaminated with a total of 4.5 mL of a liquid inoculum containing 6 log_10_ CFU of *C. difficile*, *B* *. atrophaeus* *, B. thuringiensis*, or *B. subtilis* spores. The 1.5 mL of the spore suspensions were poured into cupped hands and participants spread the inoculums over their hands below the wrists for 20 seconds. Hands were allowed to air dry for 30 seconds. The contamination procedure was repeated three times. Baseline hand contamination levels and levels after the hand disinfection procedure were measured using the glove juice method [34]. In brief, a sterile glove was donned on each hand and filled with 50 mL of Dey-Engley neutralizer solution. Gloves were secured above the wrist and hands were massaged for 60 seconds. Neutralizer was collected from the gloves, serially diluted 10-fold, and plated on CDBA media to determine *C. difficile* counts. Log_10_ reductions were calculated by subtracting log_10_ CFU recovered after hand hygiene treatment from log_10_ CFU recovered from hands without treatment (baseline).

The hand disinfection interventions included alcohol hand gel (Purell, GOJO Industries, Akron, OH), 2 mL of 0.05% triclosan liquid soap (STERIS Corporation, Mentor, OH), 70% ethanol wipe, Vashe wipe, soap and water soak, Vashe solution soak, and soap and water handwashing followed by Vashe solution soak. For the soap and water handwash, hands were rubbed vigorously with liquid soap for 20 sec, rinsed with water until soap was completely removed, and patted dry with paper towels. For the hand wiping experiments, terry cloth wipes were soaked in Vashe or 70% ethanol solution (negative control). The cloths were used to thoroughly wipe the hands for 20 seconds, either once or twice using a fresh cloth soaked in the appropriate solution. For soaking experiments, contaminated hands were immersed in 1 gallon of Vashe solution or 1 gallon of water containing 10 mL of 0.5% triclosan liquid soap (STERIS Corporation) in a sterile plastic container (13 x 12 x 6 inches) and massaged vigorously for 1 minute.

### Effectiveness of Bed Baths Using Vashe for Removal of *C. difficile* Spores from Skin

We compared the effectiveness of bed baths using Vashe solution versus nonmedicated soap and water for removal of spores from skin of patients with recently diagnosed CDI (i.e., bathing was performed within the first 4 days of CDI therapy). For bed baths, a standardized protocol was followed in which the patient’s groin, abdomen, chest, arms and hands were thoroughly wiped with a terrycloth towel soaked in the appropriate solution. The towel was soaked a second time in the solution and the test areas were wiped again. Sterile swabs (BD BBL™ CultureSwab™, Becton Dickinson) pre-moistened with Dey-Engley neutralizer (Remel Products) were used to collect cultures before and 20-30 minutes after the bed bath. The swabs were transferred to an anaerobic chamber and plated directly onto pre-reduced CDBA.

### Statistical Analysis

For hand wash experiments, the log reduction of spore contamination was determined by calculating the difference in log _10_CFU recovered from baseline and experimental groups. Mean log_10_ reductions were calculated after repeating each experiment in triplicate. Unpaired *t* tests were used to evaluate differences between the hand hygiene intervention groups. For the patient bathing study, Fisher’s exact test was used to compare the percentages of positive cultures and paired *t* tests were used to compare mean CFUs recovered before versus after bed baths.

## Results

### Impact of Organic Load on the Efficacy of Electrochemically Activated Saline

**Figure 1:**
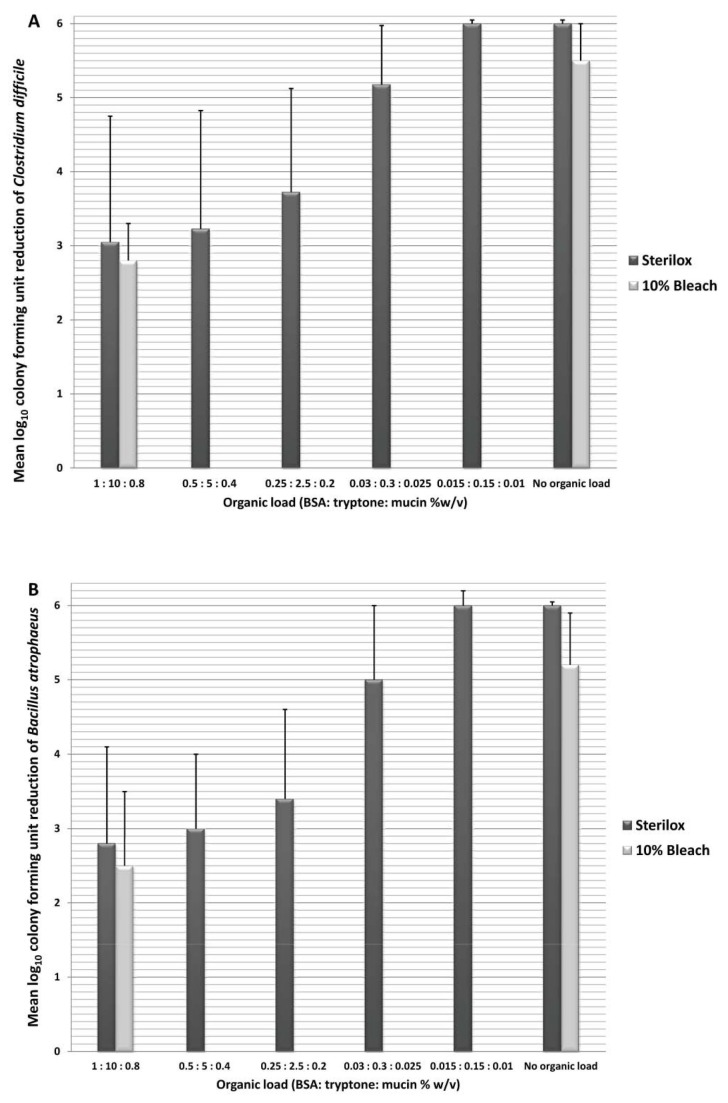
Impact of organic load on disinfection of surfaces. Mean log_10_ reduction in recovery of *Clostridium difficile* (A) and *Bacillus* *atrophaeus* (B) spores from equipment surfaces after spraying and allowing 5 minutes contact time with deionized water, Sterilox solution, or 10% household bleach, with or without a simulated organic load consisting of bovine serum albumin, tryptone, and mucin. Error bars represent standard error.

Figure 1 shows the impact of organic load on the efficacy of Sterilox and bleach for surface disinfection. Figure 1.A. and 1.B. display the reductions of *C. difficile* spores and *B* *. atrophaeus* spores respectively. There was no significant difference between reductions of *C. difficile* and *B* *. atrophaeus* spores for Sterilox or bleach (*P* = 0.8). Application of Sterilox solution on surfaces consistently resulted in a ≥6 log_10_ CFU reduction of spores in 5 minutes in the absence of organic load. The sporicidal activity of Sterilox and bleach was significantly reduced in the presence of organic load; however, even at highly saturated concentrations of organic load there was an approximately 2.5-3.0 log_10_ CFU reduction of spores after 5 minutes.

### Efficacy of Vashe for Removal of Spores from Hands

**Figure 2:**
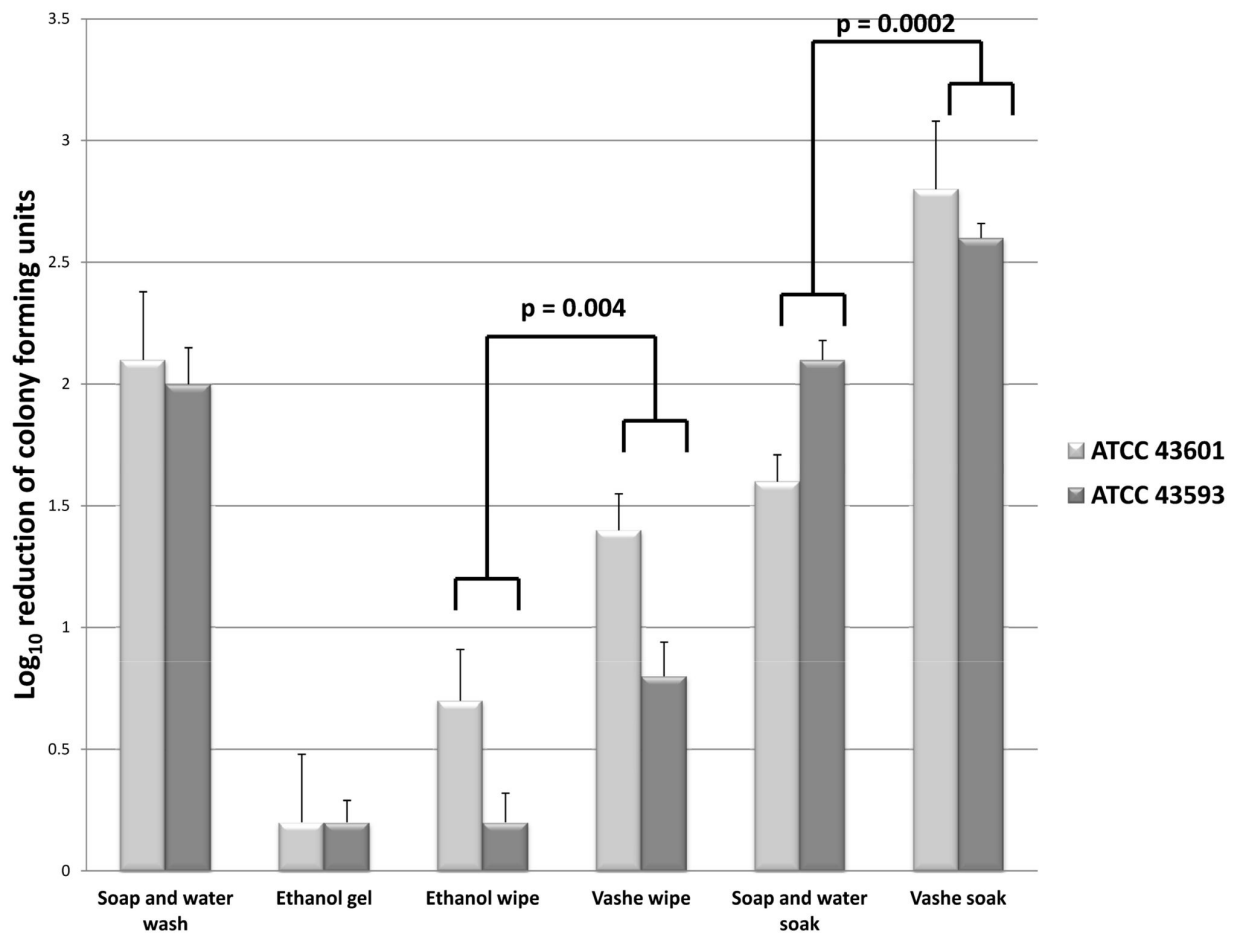
Effectiveness of Vashe for removal of *C. difficile* spores from hands. Mean log_10_ reduction in recovery of 2 strains of nontoxigenic *Clostridium difficile* from hands after performing different hand hygiene interventions. Hand hygiene interventions included alcohol hand gel, 2 mL of 0.05% triclosan liquid soap, 70% ethanol wipe, Vashe wipe, soap and water soak, and Vashe solution soak. For soaking experiments, contaminated hands were immersed in 1 gallon of Vashe solution or 1 gallon of water containing 10 mL of 0.5% triclosan liquid soap and massaged vigorously for 1 minute. Error bars represent standard error.

Figure 2 shows the efficacy of hand hygiene interventions on hands artificially contaminated with non-toxigenic *C. difficile* spores. Standard soap and water hand wash removed approximately 2 log_10_ CFU of spores from hands, whereas use of alcohol hand gel resulted in minimal reductions in spores. In comparison to wiping hands with cloths soaked in 70% ethanol (i.e., control for the mechanical removal of spores by wiping with a wet cloth), wiping with Vashe soaked cloths significantly enhanced reduction of spores by approximately 0.5 log_10_ CFU (*P* <0.01). In comparison to a soap and water soak, soaking in Vashe solution enhanced reduction of spores by 0.5 to 1.2 log_10_ CFU (*P* < 0.01). Soaking in Vashe solution also resulted in significantly greater spore removal than standard soap and water hand wash (*P* <0.01). None of the volunteers reported adverse effects related to use of the Vashe solution and no evidence of skin irritation was observed.

**Figure 3:**
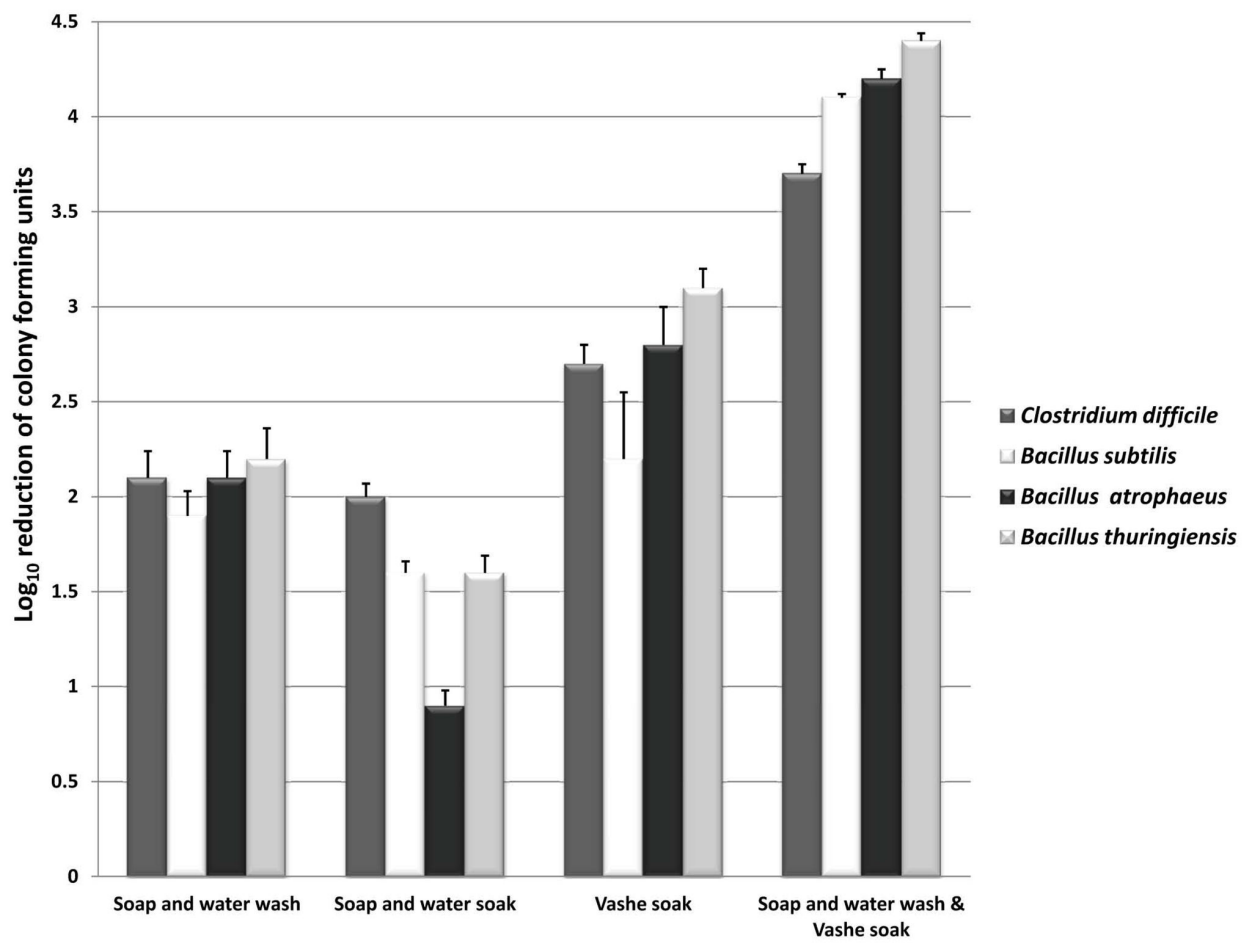
The efficacy of Vashe hand hygiene interventions for removal of *B. anthracis* surrogates and *C. difficile* spores from hands. Mean log_10_ reduction in recovery of three *Bacillus anthracis* surrogate strains and one nontoxigenic *Clostridium difficile* strain from hands after performing hand hygiene interventions. Hand hygiene interventions included soap and water hand wash, soap and water soak, Vashe solution soak, and a coupled approach consisting of a soap and water hand wash followed by Vashe solution soak. For soaking experiments, contaminated hands were immersed in 1 gallon of Vashe solution or 1 gallon of water containing 10 mL of 0.5% triclosan liquid soap and massaged vigorously for 1 minute. Error bars represent standard error.

Because organic load reduced the efficacy of electrochemically generated hypochlorous acid solutions, we hypothesized that a coupled hand hygiene intervention consisting of a standard soap and water hand wash to reduce organic load followed by soaking hands in Vashe solution would increase the efficacy of Vashe. Figure 3 demonstrates the effectiveness of the coupled hand hygiene intervention on removal of *C. difficile* (ATCC 43593) and *B. anthracis* surrogate spores artificially contaminated on hands. Consistent with previous findings, soap and water hand washing removed approximately 2 log_10_ CFU of *C. difficile* and *B. anthracis* surrogate spores. The incorportation of a Vashe soak following hand washing enhanced the reduction of spores by >1.5 log_10_ CFU compared to washing alone (*P* <0.001).

### Efficacy of Bathing with Vashe for Removal of *C. difficile* Spores from Patient Skin

**Figure 4:**
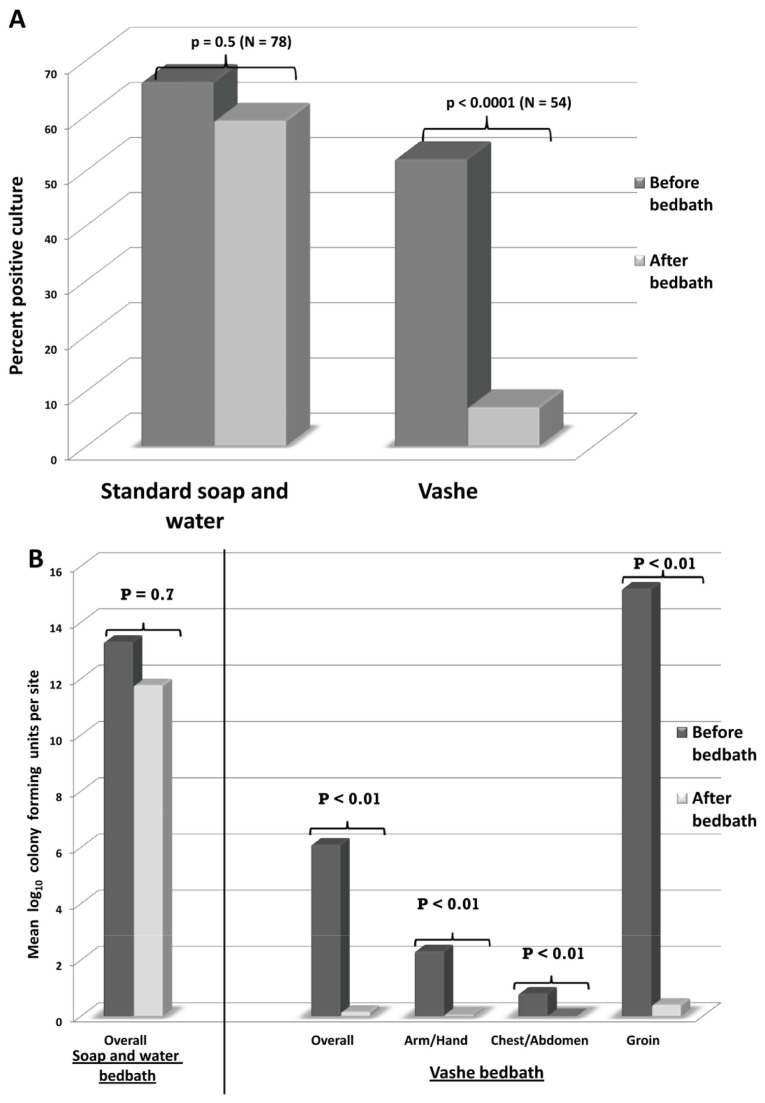
Effectiveness of bed baths using Vashe for removal of spores from skin. Comparison of soap and water versus Vashe bed baths for removal of *Clostridium difficile* spores from skin of patients with *Clostridium difficile* infection. (A) Percentage of positive skin cultures. (B) Mean colony-forming units of *C. difficile* recovered.

Figure 4A shows the proportion of positive *C. difficile* skin sites before and after bed bath for 47 CDI patients. All of the subjects were male. For bed baths with soap and water, 64% of sites on patients’ skin were positive before bathing and 57% of sites remained positive after bed baths (*P* =0.5). In contrast, Vashe bed baths resulted in a significant reduction in positive cultures from 54% of skin sites before bathing to 8% after bathing (*P* <0.0001). Figure 4B shows the mean colony forming units found on skin sites before and after bed baths. Soap and water bathing did not reduce the burden of spores on skin (range before bathing: 110-1 CFU, range after bathing: 102-1 CFU; *P* =0.7), whereas Vashe bathing significantly reduced the number of spores recovered after bathing (range before bathing: 122-1 CFU, range after bathing: 3-1 CFU; *P* <0.01). None of the patients reported any adverse effects related to bathing with the Vashe solution.

## Discussion

As has been found in previous studies, we demonstrated that alcohol hand gel was not effective in reducing levels of *C. difficile* and *Bacillus* spp. spores on hands, while soap and water hand washing reduced spore counts by about 2 log_10_ CFU [5,18,21,22]. The Vashe electrochemically generated hypochlorous acid solution enhanced spore removal from hands when applied by wiping with a wet cloth or as a one minute soak. A coupled approach consisting of a standard soap and water hand wash to reduce organic load followed by soaking hands in Vashe solution removed >3.5 log_10_ CFU of *C. difficile* and bacillus spores from hands. Moreover, bed baths using soap and water were ineffective in reducing *C. difficile* spore counts on infected patients’ skin, whereas bathing with Vashe significantly reduced both the frequency of contamination and the burden of spores on skin. There was no evidence of adverse effects due to hand washing or bathing with the solution. These findings suggest that electrochemically generated solutions containing hypochlorous acid could provide a safe and effective method to enhance removal of *C. difficile* and *Bacillus* spores from hands or skin of contaminated individuals.

In comparison to the log reduction in spore counts observed on surfaces, the hypochlorous acid-containing solutions resulted in lower reductions on hands. It is likely that the more modest efficacy at this site is related in part to the presence of organic material on skin. On surfaces, a simulated heavy organic load decreased the log reduction in spores produced by Sterilox by ~3 log_10_ CFU. We postulate that removing organic material by washing hands with soap and water contributed to the increased efficacy of the coupled approach of hand washing followed by soaking in Vashe. Additional research is needed to determine whether higher concentrations of hypochlorous acid might be more effective on skin while retaining a good safety and tolerability profile. Because the levels of spores used in laboratory studies may be several orders of magnitude greater than levels on hands of healthcare workers, there is also a need to evaluate the effectiveness of hypochlorous-acid containing solutions for hand hygiene in real-world settings.

The skin of CDI patients is often heavily contaminated with spores at the time of diagnosis [23,35]. Moreover, skin contamination often persists after diarrhea resolves and after shedding of spores in stool has been reduced to minimal levels by CDI therapy [4]. Our results suggest that bathing of CDI patients with hypochlorous acid-containing solutions might significantly reduce the burden of spores on skin. Previous studies have demonstrated that daily bathing with antimicrobial soaps such as chlorhexidine gluconate may reduce infections with vancomycin-resistant *Enterococcus* , methicillin-resistant *Staphylococcus aureus*, and *Acinetobacter baumanii* [24–26]. Similar studies are needed to evaluate the effectiveness and tolerability of daily bathing with hypochlorous acid-containing solutions as a control strategy for *C. difficile*.

Our study has some limitations. The subjects included only men from a single institution. Additional studies are needed in other patient populations. For the patient bed baths, it was not feasible to randomize subjects due to the irregular availability of fresh hypochlorous acid-containing solutions. However, baseline levels of skin contamination were similar in the standard bathing and experimental groups. Electrochemically activated saline solutions lose antimicrobial activity over time. However, commercial generators are available that produce fresh solutions for use. Future studies should be performed with commercial generators to insure peak antimicrobial activity of solutions and availability of fresh solutions for randomization of patients.

In summary, we found that a novel sporicidal electrochemically activated saline solution containing hypochlorous acid was a safe and effective method to enhance removal of *C. difficile* and *B. anthracis* surrogate spores from hands and skin of patients. Studies are needed to determine if modifications of the solutions will further increase their effectiveness and to assess whether use of these solutions will be helpful as an adjunctive method to reduce the risk for transmission of *C. difficile* or to reduce skin contamination with *B. anthracis* in an emergency setting.
